# “Do it-yourself”: Home blood pressure as a predictor of traditional and everyday cognition in older adults

**DOI:** 10.1371/journal.pone.0177424

**Published:** 2017-05-17

**Authors:** Sophie E. Yeung, Wendy Loken Thornton

**Affiliations:** Department of Psychology, Simon Fraser University, Burnaby, British Columbia, Canada; The University of Tokyo, JAPAN

## Abstract

**Background:**

Hypertension guidelines recommend home blood pressure (HBP) monitoring in adjunct to office blood pressure (OBP) for its greater reproducibility and prognostic utility in the prevention of cardiovascular outcomes, especially stroke. To date, the relationship between HBP and cognitive function remains unexplored.

**Methods:**

We examined HBP as a cognitive predictor in a multi-ethnic group of community-dwelling adults aged 60 and over (*N* = 133) using neuropsychological measures and analyzed the data using multiple regression analyses. We also employed “everyday cognition” measures that have been found to have higher prognostic utility for real-world functioning than traditional cognitive tasks.

**Results:**

Good to perfect HBP monitoring compliance over seven days was achieved by 88.7% of the participants with superior reliability (ICC≥.96) to office readings. Higher home systolic BP and pulse pressure predicted worse processing speed, executive function, and everyday cognitive function, whereas lower home diastolic BP predicted worse everyday cognition. Office readings were similarly associated with everyday cognitive function but with no other cognitive measures.

**Conclusion:**

Our findings are the first to validate HBP as a predictor of neuropsychological function in older adults beyond cognitive screening. Differential relationships among blood pressure variables and specific cognitive domains were observed. With proper standardization and training, we demonstrated that HBP can be obtained in a multi-ethnic community-dwelling older adult cohort. Our findings emphasize the importance of employing blood pressure and cognitive measures that are adequately sensitive to detect vascular-related cognitive impairment in a relatively healthy population. Implications regarding proper HBP measurement for hypertension management, cognitive health, and everyday function are discussed.

## Introduction

Hypertension is a leading risk factor for morbidity and mortality worldwide [1]. Among older adults, uncontrolled blood pressure is associated with increased risk of vascular dementia [2] and Alzheimer’s disease [3]. While mounting evidence supports that blood pressure dysregulation is a risk factor for cognitive impairment and lower quality of life [4], many inconsistencies exist within the literature. Several studies report that elevated blood pressure in mid-life predicts cognitive decline, while others demonstrate that lower blood pressure is associated with worse cognitive abilities in older adults [5–7]. Discrepant findings likely reflect varied populations, age range, and the methods used to assess blood pressure and cognition.

The majority of studies exploring blood pressure and cognition employ office or clinic blood pressure (OBP). While typical in standard practice, OBP is limited to a blood pressure “snapshot” characterized by high variability due to random (i.e., physiological and psychological state variables) and systematic error [8]. Ambulatory blood pressure (ABP) or 24-hour monitoring has the capacity to overcome multiple inadequacies of OBP and thus maintains stronger associations with cerebrovascular events [9] and cognitive function [10, 11]. However, practical limitations of ABP include economic costs and lifestyle disruption [12].

International guidelines advocate for using home blood pressure (HBP) monitoring in assessment, diagnosis and management in clinical practice and research [13–16]. Established HBP protocols optimize standardization, increase data reproducibility, and minimize factors that contribute to random error by taking readings over several days in a “real-world” environment [8]. Upon retest, a comparative study found home reliability was as high as *r* = 0.91 while office test-retest fell between *r* = 0.6–0.77 [17]. Home readings tend to be significantly lower than office readings [18–20] and correlate more closely with ABP readings [8, 21].

The utility of home readings is pronounced in older adults who demonstrate higher white-coat hypertension rates and blood pressure variability than younger adults [21, 22]. Over 50% of older outpatients attending their first visit for a cognitive evaluation are estimated to have substantial white coat hypertension [23], which could lead to overestimations of hypertensive severity [18]. Home readings also identify individuals with masked hypertension and prehypertension who are at increased risk for cognitive dysfunction and stroke [8]. Inaccurate office readings can lead to misdiagnosis and excessive blood pressure reduction that could induce cerebral hypoperfusion [12]. Furthermore, home readings predict cardiovascular and cerebrovascular morbidity and mortality better than office readings [24–27] and comparably to ABP [19]. While elevations of 10mmHg systolic and 5mmHg diastolic OBP predicted an 8% increase in risk of stroke among community-dwelling adults, the same HBP elevation was associated with a 27% and 16% increased cerebrovascular risk, respectively [26].

The association between HBP and cognitive function remains essentially unexplored, and cognitive relationships using OBP vary widely. Alterations in cerebral blood flow, or cerebral autoregulation, may lead to cerebral hypoperfusion, ischemic injury, and cognitive decline [28]. In contrast, research supporting the opposite where lower blood pressure predicted lower cognitive ability suggests that dysfunctional cerebral autoregulation induces chronic hypoperfusion and mild brain ischemia [29]. A certain blood pressure level may help ensure sufficient cerebral perfusion for optimal cognitive function but is not yet determined [30]. HBP provides a reliable ecological valid measurement that may better detect subtle cognitive change.

### Measuring blood pressure

The relationship between blood pressure and cognition is further complicated by which blood pressure variables are analyzed. In a review by the American Society of Hypertension, both elevated SBP and lower DBP in older adults were associated with increased dementia risk [28]. However, which blood pressure variable plays a more important role in predicting cognitive dysfunction remains unclear [31, 32]. Pulse pressure (PP) is used as a surrogate marker for arterial stiffness and may predict cardiovascular outcomes better than SBP [33] but continues to be an under-researched cerebrovascular risk factor [34]. Excessively high and low PP have both been linked to increased dementia risk after adjusting for SBP and DBP [35]. Of the few studies conducted, elevated PP predicted lower scores on cognitive screening [36], verbal memory, working memory, processing speed [37, 38] and theory of mind tasks [39, 40]. In a prospective study, older Chinese adults with higher office PP were more likely to develop cognitive impairment over six years [31]. To our knowledge, the current study is the first to utilize home-based SBP, DBP, and PP to uncover diverse patterns between blood pressure and cognition.

### Measuring cognition

The relationship between blood pressure and cognition is strongly determined by the cognitive tasks administered [4]. Evidence links uncontrolled blood pressure to subcortical regions vulnerable to ischemic effects, leading to declines in executive function, processing speed [32], attention and memory [41]. The few studies examining cognition using ambulatory readings typically are limited to screening tools that do not examine specific domains [11, 42]. Screening tasks are prone to ceiling effects and may lack sensitivity to detect mild cognitive changes [43]. An isolated study examining older adults found no significant relationship between HBP and Mini Mental State Examination (MMSE) scores [44], but diverse cognitive domains were not investigated. We explored HBP in relation to specific cognitive functions using various neuropsychological measures with demonstrated sensitivity to uncontrolled blood pressure.

#### Everyday cognition

An expanding area of interest is the impact of health on everyday cognition. Everyday cognitive tasks offer a means to assess real-world function by using representations of situations that individuals are more likely to encounter in their daily lives [45]. Lab-based cognitive tasks are criticized for emphasizing cognitive abilities vulnerable to aging, such as processing speed [45] and may underestimate how individuals perform in their natural environments. Everyday cognitive tasks possess greater ecological validity by requiring individuals to draw on accumulated experience and context. Everyday cognition appears to represent a distinct aspect of cognition with additive utility in predicting functional outcomes, including medication adherence, self-rated functioning, life-skills functioning, and mortality [46–49].

In the current study, we assessed everyday cognition using problem solving tasks that can be divided into well-structured problems where the initial state and desired end-state are given [45] and ill-structured problems where open-ended questions require as many safe and effective solutions as possible [50]. Ill-structured measures rely more on response fluency demands in addition to functional crystallized knowledge [51]. Previous work revealed specific associations between low OBP and decreased performance on ill-structured everyday problems in older women [52]. The current study incorporated both ill- and well-structured tasks to broadly assess everyday cognition.

### Objectives and hypotheses

We assessed whether home systolic (hSBP), diastolic (hDBP), and pulse pressure (hPP) predicted performance on traditional and everyday cognitive measures after accounting for a hypertensive diagnosis and other covariates associated with cognitive ability (i.e., demographic variables, cardiovascular risk factors, self-rated depressive and anxiety symptoms). Traditional cognitive domains typically associated with uncontrolled blood pressure (i.e., episodic memory, processing speed, and executive function) were examined using neuropsychological and everyday cognitive tasks. Office readings were used as a comparison to home readings.

We hypothesized that increased HBP would predict poorer traditional and everyday cognitive ability beyond the effects of demographic and health covariates. Since SBP and PP tend to increase more rapidly than DBP in older age [53], we predicted that hDBP would have a restricted range and that the proportion of variance in cognitive performance accounted for by hSBP and hPP would exceed that accounted for by hDBP [9, 42]. We anticipated that HBP would be more reliable than OBP and would therefore demonstrate stronger relationships with cognitive function.

In secondary analyses, linear and curvilinear blood pressure-cognition relationships were considered by examining quadratic forms of HBP and OBP. Very low and very high blood pressure may be less favourable than a median range for optimal cognitive function [54]. Lastly, age-blood pressure interactions were examined based on past evidence indicating differential cognitive relationships across diverse age ranges [55, 56].

## Materials and methods

### Participants

This study was approved by the Simon Fraser University (SFU) Research Ethics Board (dore@sfu.ca; approval number 2011s0112). Adults aged 60 and above within metro Vancouver (*n* = 133) were sought through family practice clinics, senior recreational and commercial centres, internet postings, and newspaper advertisements. Participants met the following inclusion criteria: (a) no uncorrected impairments in sensory or motor functions that might interfere with testing, (b) English fluency as determined by an acculturation measure examining language preferences [57], and (c) a minimum grade 6 education to certify that reading level was adequate for questionnaire completion. All participants provided written informed consent. Participants were screened for visual acuity with a set corrected binocular lower limit of 20/70. Exclusion criteria included a self-reported diagnosis of dementia by a physician or a score of less than 24 on the MMSE [58]; diagnosis of a major psychotic illness, concurrent terminal or neurological illness; prior major stroke; head injury defined by a loss of consciousness > 5 minutes; or current substance or alcohol use requiring treatment. At an alpha level of 0.05 and a statistical power of 0.80 to control for type 1 and type 2 error, respectively, an *n* of 133 was sufficient to detect medium and large effect sizes [59].

### Procedure

Participants attended two sessions separated by a minimum of eight days and maximum of one month. During Session #1, OBP readings were initially taken. Each participant then completed a one-hour HBP monitoring education and training session. Presentations were provided individually or in small groups of 2–3 members. Material was based on research literature and published resources available from the Canadian Hypertension Society and the Canadian Hypertension Education Program [14]. A list of guidelines regarding proper environment and variables to consider during measurements were provided to ensure standardization, as well as a monitoring log to record readings. Practice demonstrations by participants were supervised until blood pressure measurement was considered proficient. Participants were asked to monitor and record their blood pressure at home for seven consecutive days (see Blood pressure protocol below).

After completing one week of HBP monitoring, participants attended Session #2, where OBP readings were again taken and participants underwent a two-hour neuropsychological test battery. Home digital monitor memory readings were verified with written logs to ensure that participants adhered to standardized protocol. Participants were compensated $30 for time and travel expenses. Sessions took place at medical clinics and at Simon Fraser University (SFU).

#### Blood pressure protocol

Office blood pressure (OBP) was taken at the beginning of both Session #1 and #2 in sitting position on the non-dominant arm by a trained research assistant using an electronic blood pressure monitor (Model: Microlife BP 3AC1-1PC). Participants were positioned with the cuffed arm supported at the level of the heart in a quiet room [22]. One practice reading was taken to familiarize the participant with the procedure. After a five minute rest, a research assistant administered three readings separated by one-minute intervals [60]. For research-quality OBP, the last three readings from both sessions (maximum 6 readings) were averaged. For an index of casual OBP, the first reading from the cognitive testing session was used.

Home blood pressure (HBP) readings were obtained using protocol adapted from published guidelines from Canada [14], the United States [22], and Japan [15]. Participants completed seven days of monitoring using the identical blood pressure monitor used for office readings. Participants self-administered three readings on the non-dominant arm in a sitting position identical to OBP protocol following a five-minute rest and separated by one-minute intervals. Readings were taken each morning within one hour of waking and again in the evening prior to bedtime. Participants were asked to record readings and qualitative information of events that may have disrupted their readings in their monitoring log (e.g., trouble managing the machine, guideline violations). Participants were advised to avoid eating, coffee, exercise, and smoking prior to morning readings. Evening readings were to be taken at least two hours after heavy physical activity and 30 minutes after light physical activity, coffee, or smoking. Readings were measured before taking medication and after micturition. Participants were further advised to avoid measurements if uncomfortable, cold, or in great pain. A maximum of 21 morning and 21 evening readings were obtained. No participants met criteria for missing blood pressure data (i.e., OBP with < 4 readings, or HBP with <12 morning or <12 evening readings).

#### Cognitive and questionnaire protocol

All measures were administered by trained research assistants during Session #2. Participants completed a health questionnaire addressing demographics variables, medications, and information on medical illnesses and treatment. Pill bottles or pharmacy receipts were used to verify medication use. Participants required a self-reported physician’s diagnosis to be considered hypertensive. Of the 52.6% of participants who provided access to their medical records, 91.4% show consistency between self-report and prescription documentation. The Center for Epidemiologic Studies Depression Scale [CES-D] [61] was used to measure self-reported *depressive symptoms*. The Multidimensional Anxiety Questionnaire [MAQ] [62] measured self-reported *anxiety symptoms*.

The MMSE [58] was used to assess *global mental status*. In terms of *Executive Function*, the Trail Making (condition 4) and Color Word Interference (condition 3) subtests were taken from the Delis-Kaplan Executive Function System [D-KEFS] [63] to measure mental flexibility and response inhibition, respectively. Task latencies (seconds) were used as outcome measures for these items. The D-KEFS Verbal Fluency subtests assessed both semantic (ability to name animals) and letter (ability to initiate words beginning with F, A, and S) fluency in one minute, respectively, which were then totalled. For *Processing Speed*, the Wechsler Adult Intelligence Scale-III (WAIS-III) Digit Symbol Coding task [DSC] [64] was administered with the total correctly matched items as the outcome measure. *Episodic Memory* was assessed using the California Verbal Learning Test-2^nd^ Edition [CVLT-II] [65]. Words recalled across Trials 1–5, Short-Delay Free Recall [SDFR], and Long-Delay Free Recall [LDFR] were examined.

Three measures were employed to assess everyday cognition. The Everyday Problem Solving (EPS) test consisted of eight interpersonal vignettes derived from previous literature [50, 66, 67] and applied in various older adult studies [46, 48, 51, 52, 57, 68]. Problems are “ill-structured” to allow for generation of multiple responses (e.g., “A person who lives alone wants to see her children more frequently. What should she do?”), and the number of safe and effective solutions generated was used as the outcome measure. Inter-rater reliability based on this sample was very high (*r* = 0.94). The Everyday Problems Test (EPT) is a well-structured task that examines problem-solving across daily living tasks [69]. Participants are shown a naturalistic stimulus (e.g., recipe, medication prescription) and are asked related short-answer questions. A shortened version was administered to minimize fatigue with a total outcome score of 0–36. Psychometric test properties indicate strong internal consistency and test-retest reliability (*r* > .83). The Everyday Cognition Battery (ECB) Knowledge Questionnaire is a 30-item multiple choice task assessing functional knowledge related to nutrition, finances, and medication [45, 51]. The total score was used as the outcome measure.

### Statistical analyses

For HBP readings, the first of seven measurement days and the first of each triplicate reading were excluded following published recommendations to ensure reliability [22]. Mean hSBP and hDBP were derived by averaging the remaining 12 morning and 12 evening readings, exceeding standards set by prior studies and HBP protocol [12, 22]. Mean oSBP and oDBP readings were calculated by averaging the last three office readings from both sessions. Pulse pressure was calculated by subtracting average DBP from average SBP.

Home blood pressure monitoring compliance was based on the number of readings completed, accuracy achieved according to a written recording log, and the presence of guideline violations based on recording accuracy. To account for systematic error, the intra-class correlation (ICC) using a one way ANOVA model (random factor: participant) following the absolute agreement definition [70, 71] was calculated by comparing mean day-to-day HBP readings of (a) day 2 vs. day 3, (b) day 3 vs. day 4, (c) day 4 vs. day 5, (d) day 5 vs. day 6, and (e) day 6 vs. day 7, and averaged as an overall ICC value for hSBP and hDBP, respectively. ICC values falling above 0.75 were considered highly reliable [72].

#### Data reduction

To reduce the number of variables in our models, we meaningfully combined highly correlated cognitive measures by conducting an Exploratory Factor Analysis (EFA) [73, 74] that resulted in four factors with eigenvalues exceeding 1.0 (S1 Table). The first factor “Memory” consisted of high loadings from episodic memory scores (CVLT-II Trial 1–5, SDFR, and LDFR). The second factor “Everyday Cognition” included two well-structured everyday cognitive tasks (EPT and ECB). The third factor “Ideational Fluency” was composed of letter fluency, animal fluency, and EPS fluency score. The fourth factor “Speed/Executive Function” had high loadings on measures of processing speed (WAIS-III DSC) and timed executive function tasks (D-KEFS Trail Making and Color-Word Inhibition). Based on factor loading criteria [75], all cognitive measures produced fair (≥.45) to excellent loadings (≥.71) on only one factor, with the exception of Trail Making that cross-loaded on Factor 2 and 4 (S2 Table).

#### Regression analyses

Hierarchical multiple linear regressions (MLR) were used to address the prediction that increased HBP would predict poorer traditional and everyday cognition beyond a hypertensive diagnosis, demographic and health covariates. Each cognitive factor score was examined as an outcome variable in main analyses. Home-based variables were analyzed separately from office-based variables to reduce error from essential multicollinearity. Only demographic and health variables correlated with cognitive performance at an a priori cut-off of *p* < .01 and that were significant predictors when included with other covariates were analyzed in their respective main regression models. Ethnicity was collapsed into a dichotomous variable (Caucasian vs. non-Caucasian), with East Asian and remaining participants grouped together due to the small number of non-Caucasian participants. Age was included to test for age-blood pressure interaction effects. Systolic and diastolic readings were examined as independent variables. Pulse pressure was examined as a unique predictor of cognitive performance in a separate model.

Demographic and health factors were entered on the first block, followed by blood pressure variables on the second block, and interaction variables on the third block. A hypertensive diagnosis was considered on the first block but was not significantly associated with any cognitive factor and dropped from final models. Curvilinear relationships were examined by including quadratic forms of blood pressure variables that predicted cognitive function in the main analyses. Moderation effects were analysed by including age X blood pressure interactions as predictors. Continuous predictors involved in interaction or curvilinear analyses were centered to reduce non-essential collinearity. Assumptions of MLR were verified prior to running analyses. To address extreme non-normality or heteroscedasticity of residuals resulting from count dependent variables, Poisson Regression analyses were considered [76] but were not required given the linear nature of the data.

## Results

### Preliminary analyses

#### Blood pressure compliance and readings

Of the 133 participants, 84.2% completed all 42 readings. Perfect compliance (i.e., no missed or incorrect recordings) was achieved by 51.9% of participants, while 36.8% achieved good compliance (>80% of readings completed, ≤5 incorrect recordings, no major or minor guideline violations). In total, 88.7% followed HBP procedures with no guideline violations.

Means and standard deviations for blood pressure variables are provided in Table 1. Using paired sample t-tests, mean hSBP was slightly lower than mean oSBP (t (132) = -2.2, p < .05 two-tailed), though this difference was not as pronounced as typically found in previous studies [20, 23]. Mean hPP was slightly lower than mean oPP (M = 52.7, SD = 9.5, t (132) = -2.0, p < .05 two-tailed). No significant difference was found between home and office DBP. As expected, mean oSBP, oDBP, and oPP were significantly lower than first-reading oSBP1 (t (132) = -10.7, p < .001), oDBP1 (t (131) = -6.7, p < .001), and oPP1 (t (131) = -8.4, p < .001), respectively, by an average of 10mmHg systolic, 3.5mmHg diastolic, and 6mmHg in PP.

**Table 1:** Demographic, blood pressure, and health characteristics.

	Participants (n = 133)			Effect Size ^a^		
	All	Hypertensive	Non-Hypertensive			
Characteristics	N = 133	n = 71	n = 62	Cohen’s *d*	Phi coefficient φ	Cramer’s V
Age (years)	70.2 (6.5)	70.3 (6.5)	70.2 (6.4)	.016		
Range	60–88	60–88	60–86			
Gender (% female)	54.9	49.3	61.3		-.120	
Ethnicity (%)						.266**
Caucasian	57.1	45.1	71.0			
East Asian	36.1	45.1	25.8			
Other	6.8	9.9	3.2			
ESL (%)	38.3	47.9	27.4		.210*	
Education (years)	14.6 (2.6)	14.2 (2.5)	15.0 (2.7)	-.31		
CES-D	7.1 (7.6)	7.6 (7.5)	6.6 (7.7)	.13		
MAQ	55.6 (12.9)	57.9 (13.5)	53.0 (11.8)	.39*		
BMI	24.9 (3.6)	25.4 (3.7)	24.3 (3.4)	.32		
hSBP (mmHg)	123.0 (12.0)	126.3 (10.2)	119.3 (12.8)	.60+		
Range	96–154	100–154	96–150			
hDBP (mmHg)	72.3 (8.5)	73.3 (8.6)	71.2 (8.4)	.25		
Range	48–97	48–97	52–91			
hPP (mmHg)	50.7 (8.5)	52.9 (8.1)	48.9 (8.6)	.48+		
Range	35–70	35–70	37–71			
oSBP (mmHg)	124.8 (13.5)	127.8 (13.2)	121.3 (13.2)	.49**		
Range	91–158	97–158	91–151			
oDBP (mmHg)	72.1 (8.9)	72.9 (9.0)	71.2 (8.7)	.19		
Range	47–98	47–95	56–98			
oPP (mmHg)	52.7 (9.5)	55.0 (9.7)	50.1 (8.6)	.53**		
Range	34–77	35–77	34–67			
oSBP1 (mmHg)	134.4 (18.19)	138.3 (16.2)	129.8 (19.3)	.48**		
Range	92–191	101–191	92–177			
oDBP1(mmHg)	75.6 (10.07)	76.5 (10.2)	74.6 (9.8)	.20		
Range	47–108	47–108	59–95			
oPP1 (mmHg)	58.8 (13.1)	61.8 (12.1)	55.3 (13.4)	.51**		
Range	33–92	38–92	33–90			
Type 2 Diabetes (%)	11.3	16.9	4.8		.190	
High cholesterol (%)	53.4	70.4	33.9		.366+	
Cardiovascular disease (%)	11.3	12.7	9.7		.047	
Myocardial infarction (%)	4.5	5.6	3.2		.058	
Current smoker (%)	6.8	8.4	4.8		.072	
Alcohol Use (yes %)	48.1	43.7	53.2		-.095	

Means and standard deviations are presented as *M (SD)*. CES-D = Centre for Epidemiological Studies-Depression Scale. MAQ = Multidimensional Anxiety Questionnaire. BMI = Body Mass Index. Blood pressure variables: hSBP = home systolic blood pressure; hDBP = home diastolic blood pressure; hPP = home pulse pressure; oSBP = mean office systolic blood pressure; oDBP = mean office diastolic blood pressure; oPP = mean office pulse pressure; oSBP1 = first-reading office systolic blood pressure; oDBP1 = first-reading office diastolic blood pressure; oPP1 = first-reading office pulse pressure.

^a^ Effect sizes for group differences were examined using phi coefficients (φ) for dichotomous categories with Yates Continuity Correction, Fisher‘s Exact Probability test for analyses containing cells with less than 5 participants, and Cramer’s V for variables with more than 2 categories. Effect sizes for continuous variables were examined using Cohen’s *d* in conjunction with independent-sample t-tests for group mean comparisons. Significance levels are indicated by *p < .05, ** p < .01, + p < .001.

#### Blood pressure reliability

Large Pearson correlations between mean home and office readings were found regarding SBP (*r* = .74, *p* < .001), DBP (*r* = .75, *p* < .001), and PP (*r* = .81, *p* < .001). Large correlations were found between mean and first-reading OBP (SBP *r* = .83; DBP *r* = .80; PP *r* = .78) and between home and first-reading OBP (SBP *r* = .70; DBP *r* = .72; PP *r* = .69), all falling at *p* < .001.

High test-retest reliability was observed for hSBP (*r* = .85) and hDBP (*r* = .87) when test-retest correlation values were averaged across days. Large test-retest correlations between mean OBP readings were similarly observed for systolic (*r* = .65) and diastolic readings (*r* = .73) but were comparatively lower than home reliability (all falling at *p* < .01). Large intra-class correlations were demonstrated for hSBP (ICC_1,5_ = .96, 95% C.I. [.95-.97]) and hDBP readings (ICC_1,5_ = .97, 95% C.I. [.97-.98]), reflecting notably high HBP consistency and reproducibility across readings. Within-class reliability of office readings produced large but attenuated ICC values for oSBP (ICC_1, 2_ = .78, 95% C.I. [.70-.85]) and oDBP (ICC_1, 2_ = .84, 95% C.I. [.78-.89]).

#### Demographic, health, and cognitive characteristics

Sample characteristics as well as comparisons between hypertensive and non-hypertensive groups are summarized in Table 1. The hypertensive group (53.4%) consisted of individuals with a self-reported physician’s diagnosis of hypertension who were taking anti-hypertensive medications (*n* = 66) and those with a hypertensive diagnosis confirmed through medical records but not taking anti-hypertensive medications (*n* = 5). The non-hypertensive group included participants without a hypertensive diagnosis and who were not taking anti-hypertensive medications. A small to medium effect size was found with regard to ethnicity (*χ2* [1, n = 133] = 9.44, *p* = .009) and ESL status (*χ2* [1, n = 133] = 5.87, *p* = .015). The non-hypertensive group consisted of more Caucasian individuals with English as a first language than the hypertensive group, which showed a higher prevalence of East Asian adults. The hypertensive group had a greater proportion of individuals with high cholesterol (*χ2* [1, n = 133] = 17.77, *p* = .001). Individuals with a hypertensive diagnosis had higher SBP and PP readings at home and in office (mean and first-reading) compared to non-hypertensive participants. No significant group differences were found regarding DBP readings.

Mean cognitive performances including hypertensive vs. non-hypertensive group comparisons are provided in Table 2. Overall high mean MMSE scores (≥28/30) were found across groups as expected in a relatively healthy community-dwelling sample. Performance across neuropsychological and everyday cognitive tasks was consistently higher in the non-hypertensive group. Medium effect sizes for higher performance in the non-hypertensive group were found on measures of Animal Fluency, EPS, and ECB total score. Modest performance differences (based on small effect sizes) between groups were found across most other cognitive measures with trends for slightly better performance in the non-hypertensive group.

**Table 2:** Mean participant performance across cognitive measures.

Cognitive Measure	All Participants (N = 133)	Hypertensive (n = 71)	Non-Hypertensive (n = 62)	Cohen’s *d* ^a^
MMSE (0–30)	28.5 (1.4)	28.4 (1.5)	28.7 (1.4)	-.21
CVLT-II Trials 1–5 (0–80)	45.6 (10.0)	44.6 (10.5)	46.8 (9.3)	-.22
CVLT-II Short Delay Free Recall	9.3 (3.0)	9.0 (3.2)	9.7 (2.7)	-.24
CVLT-II Long Delay Free Recall	9.9 (3.1)	9.6 (3.3)	10.2 (2.7)	-.20
WAIS-III Digit Symbol Coding	58.3 (14.1)	56.5 (13.9)	60.4 (14.2)	-.28
D-KEFS Trail Making Number-Letter Switching (seconds)	103.7 (38.8)	106.2 (40.1)	100.9 (37.4)	.14
D-KEFS Color-Word Interference Inhibition (seconds)	65.8 (17.1)	66.8 (17.5)	64.6 (16.8)	.13
D-KEFS Letter Fluency	39.5 (12.2)	10.9 (3.3)	11.6 (4.0)	-.19
D-KEFS Animal Fluency	18 (4.8)	17.1 (4.1)	19.0 (5.3)	-.40*
Everyday Problem Solving	32.9 (13.4)	30.0 (11.3)	36.2 (15.0)	-.47**
Everyday Problems Test (0–36)	25 (6.3)	24.3 (6.7)	25.8 (5.7)	-.24
Everyday Cognitive Battery Knowledge (0–30)	22.8 (2.7)	22.1 (2.7)	23.5 (2.5)	-.54**

Raw scores are presented as means and standard deviations *M (SD)*. MMSE = Mini Mental Status Examination. CVLT-II = California Verbal Learning Test-II. WAIS-III = Wechsler Adult Intelligence Scale-Third Edition. D-KEFS = Delis-Kaplan Executive Function System. Trail Making and Color-Word subtests are scored in seconds, where higher score indicates worse performance.

^a^ Effect sizes comparing Hypertensive and Non-Hypertensive group means were examined using Cohen’s *d* in conjunction with independent-sample t-tests for group mean comparisons as indicated by *p < .05, ** p < .01, + p < .001.

### Main regression analyses

With regard to Everyday Cognition, inverse relationships were observed among the blood pressure variables where increased hSBP (95% C.I. = -.031 to -.001) and decreased hDBP (95% C.I. = .003-.047) were significantly associated with worse performance (Table 3). Together, 36.4% of variance in Everyday Cognition was accounted for by the full model (*R*^*2*^ = .364, F [6, 120] = 11.47, *p* < .001). When PP was examined as a separate predictor, there was a negative trend where increased hPP was associated with lower Everyday Cognition, but this relationship did not reach statistical significance.

**Table 3:** Multiple regressions examining effects of demographic variables, home blood pressure (HBP), and cognitive factors.

	**Everyday Cognition**				
**Predictor**	***B***	***S*.*E*.**	**β**	***P-value***	***ΔR***^***2***^ ^**a**^
Age	-.031	.010	-.235	.003**	
Education	.084	.024	.258	.001+	
Ethnicity	.575	.129	.340	.001+	
CES-D	-.039	.008	-.349	.001+	.336+
*Systolic & diastolic BP*					
hSBP	-.015	.008	-.220	.04*	
hDBP	.035	.011	.254	.02*	.028
Age X hSBP	.001	.001	.032	.77	
Age X hDBP	.002	.001	.145	.19	.026
*Pulse Pressure*					
hPP	-.015	.008	-.147	.06	.019
Age X hPP	.001	.001	-.008	.91	.000
	**Speed/Executive Function**				
Age	-.043	.010	-.348	.001+	
Education	.068	.025	.219	.008**	.194+
*Systolic & diastolic BP*					
hSBP	-.017	.008	-.247	.04*	
hDBP	.016	.011	.168	.16	.027
Age X hSBP	-.001	.001	-.069	.41	.004
*Pulse Pressure*					
hPP	-.017	.008	-.175	.04*	.026*
Age X hPP	-.001	.001	-.069	.41	.004

Blood pressure variables: hSBP = home systolic blood pressure; hDBP = home diastolic blood pressure; hPP = home pulse pressure. CES-D = Centre for Epidemiological Studies-Depression Scale. Gender, MAQ, a hypertensive diagnosis, and high cholesterol were initially entered in the first block but did not significantly predict any cognitive factor and were removed from final analyses. For Speed/Executive Function analyses, ethnicity and CES-D scores were also entered after block one but were non-significant predictors and removed from final analyses.

^a^ Significant ***Δ***R^2^ are identified as follows: *p < .05, ** *p* < .01, + *p* < .001.

For Speed/Executive Function (Table 3), increased hSBP predicted worse performance after accounting for significant covariates (95% C.I. = -.033 to -.001), though hDBP was not a significant predictor. The full model accounted for 22% of variance in Speed/Executive Function ability (*R*^*2*^ = .221, F [4,123] = 8.71, *p* < .001). In a separate analysis, increased hPP predicted lower Speed/Executive Function performance (95% C.I. = -.033 to -.001), accounting for 3% of variance beyond demographic covariates. No HBP variables emerged as significant predictors of Memory or Ideational Fluency.

Separate regression analyses were conducted with mean oSBP and oDBP as the predictors of interest. Consistent with home readings, increased oSBP (95% C.I. = -.029 to -.002) and decreased oDBP (95% C.I. = .001 to .043) were associated with worse Everyday Cognition (Table 4). The full model accounted for 36.5% of cognitive variance (*R*^*2*^ = .365, F [6, 120] = 11.52, *p* < 0.001). In a separate model, increased oPP significantly predicted lower Everyday Cognition, accounting for 2.4% of cognitive variance. No significant associations were found with the other cognitive factor scores. As such, home and mean office readings similarly predicted Everyday Cognition, but home readings also predicted Speed/Executive Function.

**Table 4:** Multiple regressions examining effects of demographic variables, office blood pressure (OBP), and cognitive factors.

	**Everyday Cognition**				
**Predictor**	***B***	***S*.*E*.**	**β**	***P-value***	***ΔR***^***2***^ ^**a**^
Age	-.031	.010	-.235	.003**	
Education	.084	.024	.258	.001+	
Ethnicity	.575	.129	.340	.001+	
CES-D	-.039	.008	-.349	.001+	.336+
*Systolic & diastolic BP*					
oSBP	-.016	.007	-.252	.02*	
oDBP	.022	.011	.230	.04*	.029
Age X oSBP	.000	.001	-.037	.74	
Age X oDBP	.002	.001	.137	.22	.012
*Pulse Pressure*					
oPP	-.014	.008	-.161	.03*	.024*
Age X oPP	.000	.001	-.025	.74	.001
	**Speed/Executive Function**				
Age	-.043	.010	-.348	.001+	
Education	.068	.025	.219	.008**	.190+
*Systolic & diastolic BP*					
oSBP1	-.010	.005	-.219	.06	
oDBP1	.008	.009	.104	.37	.025
*Pulse Pressure*					
oPP1	-.010	.005	-.162	.05*	.025*
Age X oPP1	-.001	.001	-.133	.10	.017

Blood pressure variables: oSBP = mean office systolic blood pressure; oDBP = mean office diastolic blood pressure; oPP = mean office pulse pressure; oSBP1 = first-reading office systolic blood pressure; oDBP1 = first-reading office diastolic blood pressure; oPP1 = first-reading office pulse pressure. CES-D = Centre for Epidemiological Studies-Depression Scale. Gender, MAQ, a hypertensive diagnosis, and high cholesterol were initially entered after block one but did not significantly predict any cognitive variable and were removed from final analyses. For first-reading analyses, ethnicity and CES-D were also entered after block one but were non-significant predictors and removed from final analyses.

^a^ Significant ***Δ***R^2^ are identified as follows: * *p* < .05, ** *p* < .01, + *p* < .001.

As an index of casual OBP, we explored whether office readings as measured by the first blood pressure reading (OBP1) predicted cognitive performance (Table 4). We stipulated that OBP1 would be more reflective of blood pressure taken during routine clinical practice where standardized protocol is rarely followed [77]. Increased oPP1 was associated with worse Speed/Executive Function performance (95% C.I. = -.02 to -.001) following entry of significant demographic covariates. Otherwise, first-reading office measurements were not associated with the other cognitive factor scores.

#### Secondary analyses

Curvilinear relationships between blood pressure and cognitive ability were analyzed by adding quadratic forms of HBP, OBP, and OBP1 to their respective main effects regression models. Quadratic variables did not predict a significant proportion of variance beyond the original variables in any model; therefore, a linear model was the best fit in qualifying the association between blood pressure and cognitive ability. Next, we explored whether an interaction between age and blood pressure (age X BP) could account for additional variance in cognitive performance. Home blood pressure variables that held significant relationships with specific cognitive factor scores were examined as moderators on the final step of their respective regression models. No significant interactions emerged when predicting Everyday Cognition or Speed/Executive Function (Table 3). Similarly, no age-OBP interactions emerged when added to the final step of their respective main effects models (Table 4). Such findings indicate that the association between blood pressure and cognitive function were consistent across all ages.

## Discussion

This study is the first to comprehensively examine home blood pressure (HBP) as a relevant predictor of neuropsychological performance among community-dwelling older adults. We first established that high home monitoring compliance rates were achievable in an elderly multi-ethnic sample following a one-hour educational session. In keeping with prior literature, HBP readings were more reliable than mean OBP despite having generally similar mean readings. Aligning with our hypotheses, increased home SBP and PP predicted decreased cognitive performance on measures of processing speed, executive function, and everyday cognition, while decreased home DBP predicted poorer performance. According to a position paper from the American Society of Hypertension, elevated SBP and lower DBP increases risk of dementia in older adults [28]. Though we did not prospectively examine HBP as a predictor of dementia, our findings revealed similar hSBP and hDBP patterns in relation to decreased cognitive performance.

The combination of increased SBP and decreased DBP draws attention to the importance of pulse pressure (PP) in relation to cerebrovascular function. Elevated PP was consistently associated with worse cognitive function using home and office readings. One explanatory mechanism relates to the impact of hypertension on arterial integrity as there is a disruption in the cerebral blood vessel structure and cerebrovascular regulatory mechanisms [32]. Decreased arterial elasticity and resulting structural and functional changes can lead to impaired brain metabolism and neuronal dysfunction [34]. Neuroimaging studies similarly indicate reduced brain volume, decreased white matter integrity, and greater risk for subcortical infarcts in older adults with elevated PP [78, 79].

Alternatively, if diastolic pressure is low, cerebral blood flow will proportionately decrease to maximize vasodilation but may lead to cerebral hypoperfusion and cognitive decline [36]. In the current study, lower DBP independently predicted worse everyday cognitive function. Older adults may require mild elevations in diastolic pressure to maintain adequate cerebral perfusion [80] and optimize cognitive function. While lower blood pressure reduces dementia and stroke risk, a large proportion of supporting studies include severely hypertensive groups. The literature has not yet extended these findings to individuals with diastolic blood pressure ≤ 75mmHg [81]. Over half of our sample (68.7%) maintained blood pressures below this range. It is possible that participants with elevated PP and decreased DBP represent an at-risk population.

Findings raise important implications for blood pressure treatment in older adults. Using data from the Hypertension in the Very Elderly Trial, individuals with exceedingly low or high DBP had greater dementia incidence [82]. The majority of our hypertensive participants (93%) were medicated, and it is possible that blood pressure in certain individuals was medically over controlled. As increased SBP and DBP can produce different or inverse cerebrovascular effects, blood pressure parameters may need to be treated differently. Research exploring the ideal goal SBP, DBP, and PP for aging cohorts may prove useful in establishing treatment protocol that optimizes cognitive function.

### Cognitive performance

We employed a neuropsychological test battery to identify whether HBP showed differential relationships with specific cognitive domains. Extending findings from office- and ambulatory-based research, results revealed novel relationships among HBP (SBP and PP), processing speed, and executive function. Impaired processing speed has been consistently found across vascular illness populations with hypertensive comorbidity [83]. Results are also in keeping with a recent scientific statement from the American Heart Association, indicating that slowed speed and executive dysfunction are most commonly associated with hypertension [32]. Current findings using HBP support the notion that deep subcortical and white matter circuits, which are critical for these cognitive functions, may be selectively impacted. Of note, office readings did not emerge as significant predictors of these abilities. When standardized procedures are followed, home readings may be more sensitive to subtle hypertension-related cognitive impairment by way of increased reliability.

Another unique aspect of the current study was the inclusion of everyday cognitive tasks requiring both fluid problem-solving and crystallized knowledge. Increased SBP and decreased DBP predicted everyday cognitive ability using both home and office readings. Performance on these everyday cognitive measures may indicate subtle cognitive decrements that may not be detected by cognitive screening measures, particularly in healthy populations without dementia. Importantly, the influence of processing speed was limited, as all everyday cognitive tasks were untimed. Everyday cognition may represent a diverse aspect of cognitive function beyond speed that is also sensitive to cerebrovascular pathology.

Associations with vascular risk factors provide impetus for everyday cognitive tasks in clinical practice where real-world function cannot easily be assessed. Functional implications for health management are raised for aging individuals with uncontrolled blood pressure where prevalent issues related to daily living include complex medical regimens, post-retirement financial management, and adjustment to aging. Future studies are required to elucidate the relationships among blood pressure, everyday cognition, dementia risk, and objective functional outcomes, such as medication adherence.

### Home vs. office blood pressure

The present study offers novel comparisons between home and office readings when predicting cognitive performance. We analyzed two types of OBP with varying levels of standardization: 1) research-quality mean OBP composed of multiple readings over two sessions to represent ideal office conditions, and 2) the initial OBP reading alone. Upon comparison, HBP emerged as the strongest consistent predictor of several cognitive domains (everyday cognition, processing speed, executive function), followed by research-quality OBP (everyday cognition), and lastly first-reading OBP.

It is interesting to note that participants achieved highly reliable readings at home after attending a one-hour education session. The pitfalls of OBP are well-recognized in the medical community, where concerns such as reduced reliability, artificially higher readings, and inflated error can outweigh the benefits of time efficiency [8]. These clinical considerations can contribute to inconsistent associations with cognitive function as seen in the literature, and at worse, clinical misdiagnosis and inappropriate drug therapy. A medical movement toward achieving “real-world” home readings will have important implications for research examining vascular risk factors for cognitive decline. With appropriate education, HBP can be a valuable adjunct to office readings through better detection of subtle cognitive and everyday functional abilities that otherwise may be missed. Nonetheless, in cases where resources for obtaining HBP are not available, highly standardized OBP protocol taken over multiple sessions may be sufficient to obtain reasonably reliable readings sensitive to certain cognitive outcomes.

### Limitations and future directions

These findings should be considered in light of certain limitations. First, we were not able to assess the direction of the association between blood pressure and cognitive performance over time due to the cross-sectional nature of this study. Longitudinal research exploring blood pressure as a prognostic risk factor would be useful to determine causal links between HBP and dementia. Second, the majority of our hypertensive participants were medically treated. One may expect the impact of antihypertensive medication to increase the level of blood pressure control, thus diluting the relationship between blood pressure and cognitive performance. Based on extant research, the benefits of antihypertensive treatment on cognitive performance remain unclear, and antihypertensive medication may impact cognitive functioning depending on the medication class [28, 32]. Larger sample studies may help determine whether participants who are not medicated or those with uncontrolled hypertension show diverse cognitive relationships. Additionally, if we had adopted a strict statistical procedure for multiple comparisons (e.g., Bonferroni correction), some analyses would not have met statistical significance. However, the consistent relationships blood pressure and cognition among home and office readings is a promising finding that aligns with prior literature and can be used as a stepping-stone for future studies. Finally, our sample size was insufficient to detect small effect sizes (i.e., *f* ^*2*^< 0.13). Certain statistical trends (p < .06) between blood pressure and cognition were observed but did not reach statistical significance due to limited power. We were still able to detect medium and large effect sizes, thereby providing groundwork for larger-scale studies to further elucidate the relationships among age, blood pressure, and specific cognitive abilities.

## Conclusions

This study is the first to validate HBP as a predictor of cognitive performance in older adults. With proper standardization and training, we demonstrated that HBP can be obtained in a multi-ethnic community-dwelling older adult cohort. We revealed novel associations with cognitive performance that were not consistently apparent when employing office measurements. Improved reliability and ecological validity of home blood pressure provides us with greater confidence regarding our understanding of blood pressure and its impact on vascular and cognitive health. These results support the hypothesis that in older age, increased systolic and reduced diastolic BP (or increased pulse pressure) may predispose to cerebral injury and result in cognitive dysfunction, which may not be detected by global cognitive screening alone. Our findings emphasize the importance of employing cognitive measures that are adequately sensitive to detect vascular-related cognitive impairment in community-dwelling samples. Promoting HBP and its relationship with cognitive ability may encourage patients to become more actively engaged in their health management. Furthermore, such research may assist medical professionals in setting appropriate blood pressure goals for older adults in order to preserve cognitive abilities and functional independence in this growing age cohort.

## Supporting information

S1 Table Initial eigenvalues (above 1) and rotation sums of squared factor loadings for exploratory factor analysis with maximum likelihood extraction using direct oblimin rotation. (DOCX)

S2 Table Pattern and structure matrix for exploratory factor analysis with direct oblimin rotation of four factor solution of cognitive outcome variables. (DOCX)
