# Supplementary material for: “Do it-yourself”: Home blood pressure as a predictor of traditional and everyday cognition in older adults
Source: PLoS One. 2017 May 17;12(5):e0177424. doi: 10.1371/journal.pone.0177424 (PMC5435167; doi:10.1371/journal.pone.0177424)
Supplement: S1 Table — (DOCX) [file pone.0177424.s001.docx]

S1 Table. Initial Eigenvalues (above 1) and Rotation Sums of Squared Factor Loadings for Exploratory Factor Analysis with Maximum Likelihood Extraction using Direct Oblimin Rotation.

| **Component** | **Initial Eigenvalues** | | **Rotation Sums of Squared Loadings** |
| --- | --- | --- | --- |
|  | Total | % of Variance | Total |
| 1 | 4.31 | 37.07 | 3.03 |
| 2 | 1.48 | 13.42 | 2.04 |
| 3 | 1.19 | 10.81 | 2.36 |
| 4 | 1.01 | 9.20 | 1.79 |
