# Supplementary material for: “Do it-yourself”: Home blood pressure as a predictor of traditional and everyday cognition in older adults
Source: PLoS One. 2017 May 17;12(5):e0177424. doi: 10.1371/journal.pone.0177424 (PMC5435167; doi:10.1371/journal.pone.0177424)
Supplement: S2 Table — (DOCX) [file pone.0177424.s002.docx]

**S2 Table. Pattern and Structure Matrix for Exploratory Factor Analysis with Direct Oblimin Rotation of Four Factor Solution of Cognitive Outcome Variables.**

| Item | Pattern coefficients | | | | Structure coefficients | | | | Communalities |
| --- | --- | --- | --- | --- | --- | --- | --- | --- | --- |
|  | Factor 1 | Factor 2 | Factor 3 | Factor 4 | Factor 1 | Factor 2 | Factor 3 | Factor 4 |  |
| CVLT-II 1-5 | **.729** | .045 | -.150 | -.021 | **.806** | .371 | -.485 | .283 | .671 |
| CVLT-II SDFR | **.943** | -.111 | -.059 | -.008 | **.926** | .259 | -.425 | .267 | .868 |
| CVLT-II LDFR | **.938** | .092 | .140 | .067 | **.931** | .402 | -.341 | .344 | .886 |
| EPT | -.015 | **.627** | -.212 | .101 | .341 | **.754** | -.519 | .425 | .621 |
| ECB | .041 | **.611** | .008 | -.048 | .247 | **.604** | -.259 | .203 | .367 |
| Trail Making | .045 | **.409** | .007 | **.436** | .329 | **.594** | -.359 | **.609** | .521 |
| EPS | .059 | .108 | **-.496** | -.057 | .301 | .325 | **-.548** | .194 | .313 |
| Letter Fluency | .030 | -.084 | **-.811** | .003 | .359 | .284 | **-.789** | .290 | .628 |
| Animal Fluency | -.023 | .052 | **-.504** | .122 | .257 | .313 | **-.564** | .329 | .335 |
| DCS | -.005 | -.060 | -.022 | **.695** | .200 | .222 | -.260 | **.678** | .463 |
| Color Word | .041 | .020 | -.020 | **.461** | .202 | .226 | -.224 | **.490** | .244 |

CVLT-II = California Verbal Learning Test-II. LDFR = Long Delay Free Recall. SDFR = Short Delay Free Recall. WAIS-III = Wechsler Adult Intelligence Scale-III. DSC = Digit Symbol Coding. EPS = Everyday Problem Solving. EPT = Everyday Problems Test. ECB = Everyday Cognitive Battery.
